# Selective Serotonin Reuptake Inhibitor (SSRI) Antidepressants, Prolactin and Breast Cancer

**DOI:** 10.3389/fonc.2012.00177

**Published:** 2012-12-05

**Authors:** Janet E. Ashbury, Linda E. Lévesque, Patricia A. Beck, Kristan J. Aronson

**Affiliations:** ^1^Department of Community Health and Epidemiology, Carruthers Hall, Queen’s UniversityKingston, ON, Canada; ^2^Kingston, Frontenac, Lennox and Addington Public HealthKingston, ON, Canada; ^3^Population Health Branch, Saskatchewan Ministry of HealthRegina, SK, Canada; ^4^Division of Cancer Care and Epidemiology, Cancer Research Institute, Queen’s UniversityKingston, ON, Canada

**Keywords:** selective serotonin reuptake inhibitors, SSRIs, breast cancer, prolactin, antidepressants, case-control studies

## Abstract

Selective serotonin reuptake inhibitors (SSRIs) are a widely prescribed class of antidepressants. Laboratory and epidemiologic evidence suggests that a prolactin-mediated mechanism secondary to increased serotonin levels at neuronal synapses could lead to a potentially carcinogenic effect of SSRIs. In this population-based case-control study, we evaluated the association between SSRI use and breast cancer risk as a function of their relative degree of inhibition of serotonin reuptake as a proxy for their impact on prolactin levels. Cases were 2,129 women with primary invasive breast cancer diagnosed from 2003 to 2007, and controls were 21,297 women randomly selected from the population registry. Detailed information for each SSRI prescription dispensed was compiled using the Saskatchewan prescription database. Logistic regression was used to evaluate the impact of use of high and lower inhibitors of serotonin reuptake and duration of use, as well as to assess the effect of individual high inhibitors on the risk of breast cancer. Exclusive users of high or lower inhibitors of serotonin reuptake were not at increased risk for breast cancer compared with non-users of SSRIs (OR = 1.01, CI = 0.88–1.17 and OR = 0.91, CI = 0.67–1.25 respectively), regardless of their duration of use or menopausal status. While we cannot rule out the possibility of a clinically important risk increase (OR = 1.83, CI = 0.99–3.40) for long-term users of sertraline (≥24 prescriptions), given the small number of exposed cases (*n* = 12), the borderline statistical significance, and the wide confidence interval, these results need to be interpreted cautiously. In this large population-based case-control study, we found no conclusive evidence of breast cancer risk associated with the use of SSRIs even after assessing the degree of serotonin reuptake inhibition and duration of use. Our results do not support the serotonin-mediated pathway for the prolactin-breast cancer hypothesis.

## Introduction

Selective serotonin reuptake inhibitors (SSRIs) are prescribed widely for the treatment of depression as well as for the management of chronic pain, menopausal symptoms, and migraine headache prophylaxis (Bahl et al., 2003; Stone et al., 2003; McIntyre et al., 2005) with prevalence estimates of SSRI use ranging from 5–10% (Patten et al., 2005; Paulose-Ram et al., 2007). SSRIs produce their therapeutic action and adverse effects by selectively binding to the serotonin (5-hydroxytryptamine, 5-HT) transporter, thereby blocking the reuptake of serotonin into pre-synaptic neurons, which results in enhanced serotonergic function in the brain (Richelson, 2001; Ward and Azzaro, 2004). Using radioligand assays, the serotonin transporter binding potency of individual SSRIs can be classified on the basis of an agent’s dissociation constant (Kd) (Tatsumi et al., 1997; Meijer et al., 2004). A lower dissociation constant reflects a higher affinity for the serotonin transporter which is associated with higher inhibition of serotonin reuptake and therefore more elevated extracellular serotonin levels at pre-synaptic junctions (Tatsumi et al., 1997; Meijer et al., 2004). Of the SSRI antidepressants, paroxetine, sertraline, and fluoxetine are the three most potent (high) inhibitors of serotonin reuptake, while citalopram and fluvoxamine can be classified as relatively lower inhibitors (Tatsumi et al., 1997; van Walraven et al., 2001; Meijer et al., 2004; Turner et al., 2007).

*In vitro* and *in vivo* evidence suggests that serotonin modulates prolactin production by inducing the release of prolactin releasing factors (PRFs), such as vasoactive intestinal peptide (VIP) and oxytocin (OT), from the hypothalamus. In turn, these substances stimulate lactotrophs in the pituitary gland to release prolactin (Emiliano and Fudge, 2004). Though results are conflicting, several case reports and small uncontrolled studies that assessed changes in prolactin levels with oral and intravenous SSRI administration indicate that all SSRIs have the potential to cause varying increases in circulating basal prolactin to levels within and above the accepted normal range (Emiliano and Fudge, 2004; Molitch, 2005; Coker and Taylor, 2010; Madhusoodanan et al., 2010; Trenque et al., 2011). The serotonin-mediated pathway has been postulated as one mechanism to explain these observations (Emiliano and Fudge, 2004).

There is considerable animal and *in vitro* laboratory evidence to provide a biologic basis for an association between elevated prolactin levels and breast cancer development in humans (Welsch and Nagasawa, 1977; Kiss et al., 1987; Vonderhaar, 1999; Emiliano and Fudge, 2004; Harvey, 2005). For example, prolactin, as a tumor-promoter, has been shown to stimulate proliferative activity in the mammary gland, suppress apoptosis (normal process of cell self-destruction), and upregulate the BRCA1 (breast cancer 1) gene (Vonderhaar, 1999; Harvey, 2005). In addition, studies in humans also support an association between elevated prolactin levels and the subsequent development of breast cancer. A pooled analysis of three large prospective cohort studies reported a small increased risk for breast cancer in women with high normal or above normal levels of prolactin (>17.6 ng/mL) compared to women with below normal levels (≤9.8 ng/mL) (RR = 1.3, CI = 1.1–1.6, *p*-trend = 0.002) (Tworoger et al., 2007). The association was even stronger after correction for the reproducibility of prolactin measurements (RR = 1.7; CI = 1.2–2.3) and was independent of menopausal status (*p* for interaction = 0.95) (Tworoger et al., 2007).

Tworoger and Hankinson (2008) concluded that accumulating evidence suggests that prolactin plays an etiologic role in breast cancer development and recommended further assessment of breast cancer risk associated with long-term use of medications such as SSRIs that are known to influence circulating levels of prolactin. Further, a recently published meta-analysis by Cosgrove et al. (2011) reported a slight increase in the risk of breast/ovarian cancer with the use of antidepressants (OR = 1.11, CI = 1.03–1.20) and specifically SSRI use (OR = 1.07, CI = 0.99–1.51). In light of these findings, Cosgrove et al. (2011) recommended further research to examine how complex inter-relationships between serotonin, SSRIs and prolactin may impact on breast cancer risk. In addition, despite significant limitations of this meta-analysis including differences between studies in terms of exposure assessment, cancer sites and adjustment for potentially confounding factors, these results have renewed public health concerns about the potential risks associated with the use of SSRIs (Cosgrove et al., 2011).

Given the widespread use of SSRI antidepressants especially among women, further assessment of this biologically plausible association with breast cancer risk is warranted. In this population-based case-control study we evaluated the effects of SSRI use on breast cancer risk as a function of their relative degree of inhibition of serotonin reuptake, a proxy for their impact on prolactin levels, and their duration of use, as well as the potentially risk-modifying effects of menopausal status.

## Materials and Methods

### Data sources and linkages

Three administrative health databases from the province of Saskatchewan (SK), Canada were linked to conduct this study: (1) the population registry for socio-demographic information and dates of insurance coverage; (2) the Saskatchewan prescription drug plan database for outpatient prescriptions filled since the mid-1970s that are eligible for coverage by the drug plan for all Saskatchewan residents with the exception of approximately 9% of the population (primarily registered Indians); and, (3) the Saskatchewan Cancer Agency (SCA) cancer registry database for detailed information on cancer diagnoses since 1970 (Downey et al., 2005). These databases are generated by the province’s universal health insurance programs where information is available from all three databases for approximately 91% of residents (about 1 million people). Each resident is represented by a unique identifier (Health Services Number) that enables record linkage across databases over time at the level of the individual (Downey et al., 2005).

### Selection of cases and controls

The selection procedures for cases and controls have been described in detail elsewhere (Ashbury et al., 2010). In brief, the underlying study population was conceptualized as all women eligible for outpatient prescription drug benefits who, during the case accrual period of 2003–2007, were: (1) aged 28–79, (2) had prescription coverage for 10 or more consecutive years prior to their index date, and (3) had no previous cancer diagnosis in the 10 years preceding their index date, with the exception of non-melanoma skin cancer and *in situ* cervical cancer. All incident cases of primary invasive breast cancer, diagnosed between January 1, 2003 and December 31, 2007 who met these eligibility criteria were included. Ten controls per case frequency-matched on the cases’ age in 5-year groups were selected from the underlying study population using an incidence density (risk-set) sampling approach (Rothman et al., 2008). The index date for cases was the date of breast cancer diagnosis, and for controls was defined by a randomly chosen date corresponding to each 6-month sampling period.

We identified 2,130 women diagnosed with breast cancer [2,119 (99.5%) primary invasive cancers and 11 (0.5%) carcinomas *in situ*, which were retained in the analysis because of their small numbers], and 21,300 controls. Due to missing demographic data, one case and three controls were excluded leaving 2,129 cases and 21,297 controls for this analysis.

### SSRI use

For each case and control, we obtained detailed information for each SSRI prescription dispensed between either the start of their drug plan coverage or July 1, 1989 (when the first SSRI was listed on the SK Formulary), whichever was later, and their index date. Information available included drug name, strength, quantity dispensed, and dispensing date but did not include dosage prescribed (number of pills per dose and total number of doses per day), duration of the prescription, and indication for the SSRI prescription. SSRIs covered by the provincial drug plan during the study period and available without prescribing restrictions included citalopram, fluoxetine, fluvoxamine, paroxetine, and sertraline. SSRI use within a 2-year period preceding the index date was not considered to be causally associated with breast cancer development and therefore excluded from the analysis. This 2-year latency period between SSRI use and breast cancer diagnosis is supported by mathematical models of tumor growth by Moolgavkar et al. (1980) and postulated tumor-promoter effects of SSRIs on breast tumor growth in laboratory animals (Brandes et al., 1992). Further, exclusion of this 2-year SSRI exposure period prior to diagnosis was chosen to minimize reverse causality bias (when SSRI use is associated with growth of an existing breast malignancy that is not yet clinically detectable).

Paroxetine, sertraline, and fluoxetine were classified as “high” inhibitors based on their greater serotonin transporter binding potency relative to citalopram and fluvoxamine (so-called “lower” inhibitors) (Tatsumi et al., 1997; Richelson, 2001). In order to test our prolactin-breast cancer hypothesis, we examined the effects of high inhibitors independent of use of lower inhibitors. Therefore, SSRI users were divided into three main exposure categories: (1) exclusive users of high inhibitors (paroxetine, sertraline, and/or fluoxetine), (2) exclusive users of lower inhibitors (citalopram and/or fluvoxamine), and (3) users of both high and lower inhibitors (combined users). The comparator group for each analysis was women with no SSRI prescriptions dispensed during the same time period of two or more years preceding the index date.

The duration of SSRI use analysis was based on the total number of prescriptions dispensed during the etiologically relevant exposure time window of two or more years prior to index date given that the exact duration of individual prescriptions was not available. However, since SSRI prescriptions in Saskatchewan are typically dispensed to accommodate a 34-day treatment period, the total number of prescriptions dispensed approximated the number of months exposed. To evaluate the effect of duration of SSRI use on breast cancer risk, exclusive users of high and lower inhibitors were further subdivided into two clinically relevant exposure categories – recipients of 1–23 and ≥24 prescriptions. These cut-offs were chosen for several reasons. Firstly, for a carcinogenic hypothesis, it is important to define exposure in terms of long-term use, preferably years of use, as short durations are unlikely to have an important effect on breast cancer risk (Shapiro, 1989). Secondly, for the main analysis (i.e., exclusive users of high inhibitors versus non-users) our goal was to assess dose response according to the longest duration of SSRI use possible while still ensuring that the uppermost category of SSRI users included an adequate number of subjects. For users of both high and lower inhibitors (combined users), the duration cut-offs (1–23 and ≥24 prescriptions) were based on the total number of prescriptions for high inhibitors only.

We also assessed the risk for breast cancer associated with the use of individual high inhibitors. This analysis was restricted to persons who had used a single high inhibitor antidepressant agent exclusively during the study period.

### Potential confounders

Cases and controls were categorized on the basis of their age (continuous), marital status (married, single, other), place of residence status (urban, rural), and income support status (no support, low support, and high support) using yearly demographic data available (see Table 1 footnotes for details). Information related to oral contraceptive and hormone therapy use was obtained from the prescription database.

**Table 1:** **Characteristics of breast cancer cases and age-matched controls**.

Characteristic	Cases *N* = 2129	(%)	Controls *N* = 21297	(%)	Unadjusted OR^a^
	*n*		*n*		(95% CI)
**Age (years)**
Mean (SD)	60.4	(11.4)	60.3	(11.4)	
**Age categories**
<40	57	(2.7)	570	(2.7)	1.00 (Reference)
40–55	685	(32.2)	6972	(32.7)	0.98 (0.74, 1.31)
>55	1387	(65.2)	13755	(64.6)	1.01 (0.76, 1.33)
**Follow-up (years)^b^**
Mean (SD)	28.9	(3.5)	28.8	(3.7)	1.01 (0.99, 1.02)
Median (range)	30.0	(10, 32)	30.0	(10.32)	
**Marital status^c^**
Other (widowed, separated, divorced)	393	(18.5)	4386	(20.6)	1.00 (Reference)
Single	139	(6.5)	1421	(6.7)	1.09 (0.89, 1.34)
Married	1597	(75.0)	15490	(72.7)	1.15 (1.03, 1.29)
**Income support status^d^**
No income support	1542	(72.4)	15049	(70.7)	1.00 (Reference)
Low income support	507	(23.8)	5375	(25.2)	0.92 (0.83, 1.02)
High income support	80	(3.8)	873	(4.1)	0.89 (0.71, 1.13)
**Place of residence^e^**
Urban	911	(42.8)	8837	(41.5)	1.00 (Reference)
Rural	1218	(57.2)	12460	(58.5)	0.95 (0.87, 1.04)
**Oral contraceptive (OC) use^f^**
None or light use (<12 prescriptions)	1620	(76.1)	16775	(78.8)	1.00 (Reference)
Heavy use (≥12)	509	(23.9)	4522	(21.2)	1.17 (1.05, 1.29)
**Hormone therapy (HT) use^f^**
None or light use (<12 prescriptions)	1503	(70.6)	15542	(73.0)	1.00 (Reference)
Heavy use (≥12)	626	(29.4)	5755	(27.0)	1.13 (1.02, 1.24)

*OR, odds ratio(s); CI, confidence intervals*.

*^a^Frequency-matched on age in 5-year groups but not adjusted for the influence of other risk factors*.

*^b^Follow-up: number of years eligible to receive outpatient prescription drug benefits in Saskatchewan*.

*^c^Marital status: attributed to the marital state 6 years prior to index date*.

*^d^Income support status: based on yearly demographic data and assigned to one of 3 categories based on the greatest proportion of time having received income support: i.e., no income support, ≤50% of time receiving income support = low income support, and >50% of time receiving income support = high income support*.

*^e^Place of residence: based on yearly demographic data and assigned based on the greatest proportion of time spent in a given residence state: i.e., ≤50% of time in urban location (population > 100,000) = rural residence status and >50% of time as urban = urban residence status*.

*^f^OC and HT use: defined as total number of prescriptions 2 or more years prior to index date. These drugs are typically dispensed as a 2-month supply; therefore, in most cases, 12 prescriptions would correspond to a 2-year supply. All oral and injectable contraceptives were included as were all hormone therapy formulations except vaginal creams and rings*.

*Percentages may not add up to 100% due to rounding*.

Since breast cancer is a heterogeneous disease where menopausal status at diagnosis has been shown to reflect different etiologies and risk factor profiles, it is plausible that prolonged elevated levels of prolactin may affect pre- and post-menopausal breast tissue differently (Tworoger et al., 2006). We used age at index date (breast cancer diagnosis date for cases) as a proxy for determining menopausal status since the latter was not available from the data sources. Pre- and post-menopausal were defined as <55 and ≥55 years of age, respectively. Phipps et al. (2010) reported minimal differences in breast cancer incidence and detection rates when comparing the impact of simple age-based definitions of menopausal status (age 50 and age 55 as cut-offs) with complex multivariable definitions thereby providing support for the use of age as a proxy for menopausal status. In our study, age 55 was chosen as a cut-off to avoid misclassification of pre-menopausal cases as post-menopausal, since breast cancer cases are more likely to self-report a later age at menopause (Morabia and Flandre, 1992; Phipps et al., 2010). In addition, previous studies investigating the role of menopausal status on breast cancer risk have used similar age cut-offs to define menopausal status (Van Hoften et al., 2000; Tryggvadottir et al., 2002; Gonzalez-Perez and Garcia Rodriguez, 2005).

### Statistical analysis

Logistic regression was used to evaluate the impact of use of high and lower inhibitors of serotonin reuptake (SSRIs) and duration of use, as well as to assess the effect of individual high inhibitors on the risk of breast cancer using non-users of SSRIs as the comparator group for all analyses. To evaluate potential confounders, the “change in estimate” method was used with covariates retained in the final model if they changed the main exposure estimate by 10% or more (Greenland, 1989; Budtz-Jorgensen et al., 2007). No variable (age, marital status, place of residence, income support status, oral contraceptive, and hormone therapy use) met the definition of a confounder. However, we report risk estimates adjusted for all of these variables since there was no loss of precision compared to the parsimonious model. A two-sided test of interaction was used with a significance threshold of α = 0.05 to test the modifying effect of age at index date (a proxy for menopausal status) (Altman and Bland, 2003).

### Sensitivity Analysis

As non-compliance is a potential source of exposure misclassification, we repeated all analyses changing the referent category from no prescriptions to ≤1 prescription.

## Results

The study population consisted of 2,129 cases and 21,297 controls. Among the cases, approximately 75% had infiltrating ductal carcinoma, 10% lobular carcinoma, and 15% had a mix of other morphologies. Almost 44% (43.8%) presented as stage one, 36.2% stage two, 13.0% stage three, 4.6% stage four, 0.5% *in situ*, and for 1.9%, stage could not be assessed. The proportion of breast cancer cases diagnosed each year was similar across the 5-year accrual period.

Cases and controls were similar with regard to age, duration of follow-up, receipt of income support (a marker of socio-economic status), and place of residence (Table 1). However, cases were somewhat more likely to be married and to have been heavy users of oral contraceptives and/or hormone therapy than controls. These differences were controlled for in the analysis.

Table 2 shows the unadjusted and adjusted ORs and 95% confidence intervals for breast cancer risk according to the degree of serotonin reuptake inhibition for the use of SSRI antidepressants during the postulated etiologically relevant time period of two or more years prior to index date. After adjustment for determinants of breast cancer risk, exclusive users of high inhibitors were not at increased risk for breast cancer compared with non-users of SSRIs (OR = 1.01, CI = 0.88 – 1.17). Exclusive use of lower inhibitors was also not associated with an increased risk (OR = 0.91, CI = 0.67 – 1.25).

**Table 2:** **Odds ratios for breast cancer according to the degree of serotonin reuptake inhibition of selective serotonin reuptake inhibitor (SSRI) antidepressants**.

SSRI category^a^	Cases *N* = 2129	(%)	Controls *N* = 21297	(%)	Unadjusted OR^b^(95% CI)	Adjusted OR^c^ (95% CI)
	*n*		*n*		
No use^d^	1783	(83.8)	17908	(84.1)	1.00 (Reference)	1.00 (Reference)
Exclusive users of high inhibitors^e^	241	(11.3)	2381	(11.2)	1.02 (0.88, 1.17)	1.01 (0.88, 1.17)
Exclusive users of lower inhibitors^e^	44	(2.1)	482	(2.3)	0.92 (0.67, 1.25)	0.91 (0.67, 1.25)
Combined users^f^	61	(2.9)	526	(2.5)	1.17 (0.89, 1.53)	1.17 (0.89, 1.53)

*OR, odds ratio(s); CI, confidence intervals*.

*^a^SSRI (selective serotonin reuptake inhibitor) use anytime during follow-up 2 or more years prior to index date*.

*^b^Matched on age in 5-year age groups but unadjusted for the influence of other risk factors*.

*^c^Adjusted for age at index (years), marital status, income support status, residence status, and use of oral contraceptives and/or use of hormone therapy*.

*^d^“No use” (reference category) refers to women with no SSRI prescriptions dispensed 2 or more years prior to index date. Subjects may have filled prescription(s) for SSRIs during the 2 years prior to index date which was not considered to be etiologically relevant*.

*^e^“Exclusive users” of high or lower inhibitors had 1 or more SSRI prescriptions dispensed from the specific sub-group of SSRIs being analyzed*.

*^f^“Combined users” refers to users of high and lower inhibitors. Cases and controls represent number of women within the “combined users” group with 1 or more prescriptions dispensed for high inhibitors*.

*Percentages may not add up to 100% due to rounding*.

We did not observe an increase in breast cancer risk according to duration of SSRI use, regardless of the degree of serotonin reuptake inhibition (adjusted OR 1.15, CI = 0.89 – 1.50 and OR = 1.40, CI = 0.67 – 2.94 for exclusive users of ≥24 prescriptions of high and lower inhibitors, respectively). However, the number of long-term users (i.e., ≥24 prescriptions) was very small (Table 3).

**Table 3:** **Odds ratios for breast cancer according to the duration of selective serotonin reuptake inhibitor (SSRI) use**.

No. of SSRI prescriptions^a^	Cases *N* = 2129 *n*	(%)	Controls *N* = 21297 *n*	(%)	Unadjusted OR^b^ (95% CI)	Adjusted OR^c^ (95% CI)
No use^d^	1783	(83.8)	17908	(84.1)	1.00 (Reference)	1.00 (Reference)
Exclusive users of high inhibitors^e^
1–23	176	(8.3)	1818	(8.5)	0.97 (0.83, 1.14)	0.97 (0.82, 1.14)
≥24	65	(3.1)	563	(2.6)	1.16 (0.89, 1.51)	1.15 (0.89, 1.50)
Exclusive users of lower inhibitors^e^
1–23	36	(1.7)	425	(2.0)	0.85 (0.60, 1.20)	0.85 (0.60, 1.19)
≥24	8	(0.4)	57	(0.3)	1.41 (0.67, 2.96)	1.40 (0.67, 2.94)
Combined users^f^
1–23	47	(2.2)	377	(1.8)	1.25 (0.92, 1.70)	1.26 (0.93, 1.72)
≥24	14	(0.7)	149	(0.7)	0.94 (0.54, 1.64)	0.94 (0.54, 1.64)

*OR, odds ratio(s); CI, confidence intervals*.

*^a^SSRI (selective serotonin reuptake inhibitor) use anytime during follow-up 2 or more years prior to index date where the number of prescriptions dispensed approximates the number of months exposed*.

*^b^Matched on age in 5-year age groups but unadjusted for the influence of other risk factors*.

*^c^Adjusted for age at index (years), marital status, income support status, residence status, and use of oral contraceptives and/or use of hormone therapy*.

*^d^“No use” (reference category) refers to women with no SSRI prescriptions dispensed 2 or more years prior to index date. Subjects may have filled prescription(s) for SSRIs during the 2 years prior to index date which was not considered to be etiologically relevant*.

*^e^“Exclusive users” of high or lower inhibitors had 1 or more SSRI prescriptions dispensed from the specific sub-group of SSRIs being analyzed*.

*^f^“Combined users” refers to users of high and lower inhibitors: subgroups represent number of prescriptions for high inhibitors only*.

*Percentages may not add up to 100% due to rounding*.

The risk of breast cancer associated with the use of SSRI antidepressants was independent of menopausal status, measured as age at index, with no risk increase observed for either users of high inhibitors (adjusted OR = 0.92, CI = 0.73–1.17 and OR = 1.07, CI = 0.90–1.28 for women <55 years and ≥55 years of age, respectively; *p* = 0.24 for test of interaction) or lower inhibitors (adjusted OR = 1.00, CI = 0.59–1.69 and OR = 0.87, CI = 0.59–1.28 for women <55 years and ≥55 years of age respectively; *p* = 0.62 for test of interaction) (Table 4).

**Table 4:** **Odds ratios for breast cancer according to total use of selective serotonin reuptake inhibitors (SSRIs) by menopausal status**.

SSRI category^c^	<55 years of age^a^						≥55 years of age^a^						*P* value interaction^b^
	Cases *N* = 692 *n*	(%)	Controls *N* = 6919 *n*	(%)	Unadjusted OR^d^ 95% CI	Adjusted OR^e^ 95% CI	Cases *N* = 1437 *n*	(%)	Controls *N* = 14378 *n*	(%)	Unadjusted OR^d^ 95% CI	Adjusted OR^e^ 95% CI	
No use^f^	566	(81.8)	5626	(81.3)	1.00 (Reference)	1.00 (Reference)	1217	(84.7)	12282	(85.4)	1.00 (Reference)	1.00 (Reference)
Exclusive users of high inhibitors^g^	85	(12.3)	927	(13.4)	0.91 (0.72, 1.16)	0.92 (0.73, 1.17)	156	(10.9)	1454	(10.1)	1.08 (0.91, 1.29)	1.07 (0.90, 1.28)	0.24
Exclusive users of lower inhibitors^g^	16	(2.3)	160	(2.3)	0.99 (0.59, 1.67)	1.00 (0.59, 1.69)	28	(2.0)	322	(2.2)	0.88 (0.59, 1.30)	0.87 (0.59, 1.28)	0.62
Combined users: users of high and lower inhibitors^h^	25	(3.6)	206	(3.0)	1.21 (0.79, 1.84)	1.26 (0.82, 1.93)	36	(2.5)	320	(2.2)	1.14 (0.80, 1.61)	1.13 (0.79, 1.60)	0.70

*OR, odds ratio(s); CI, confidence intervals*.

*^a^Age at index date used as a proxy for menopausal status*.

*^b^p value for 2-sided test for interaction (Altman and Bland, 2003)*.

*^c^SSRI (selective serotonin reuptake inhibitor) use anytime during follow-up 2 or more years prior to index date*.

*^d^Matched on age in 5-year age groups but unadjusted for the influence of other risk factors*.

*^e^Adjusted for age at index (years), marital status, income support status, residence status, and use of oral contraceptives and/or use of hormone therapy*.

*^f^“No use” (reference category) for each analyses refers to women with no SSRI prescriptions dispensed 2 or more years prior to index date. Subjects may have filled prescription(s) for SSRIs during the 2 years prior to index date which was not considered to be etiologically relevant*.

*^g^“Exclusive users” of high or lower inhibitors had 1 or more SSRI prescriptions dispensed from the specific sub-group of SSRIs being analyzed*.

*^h^“Combined users” refers to users of high and lower inhibitors. Cases and controls represent number of women within the “combined users” group with 1 or more prescriptions dispensed for high inhibitors*.

*Percentages may not add up to 100% due to rounding*.

Table 5 presents the risk estimates for breast cancer for exclusive users of individual high inhibitors of serotonin reuptake (i.e., paroxetine, sertraline, or fluoxetine) compared with non-users of SSRI antidepressants. Short-term use of any of the high inhibitors was not associated with a significantly increased risk of breast cancer. Although long-term use of sertraline was associated with an elevated OR that was approaching statistical significance (adjusted OR = 1.83, CI = 0.99–3.40), this analysis was based on only 12 exposed cases.

**Table 5:** **Odds ratios for breast cancer according to total “exclusive use” of each individual high inhibitor antidepressant agent**.

No. of SSRI prescriptions^a^	Cases^b^ N = 2129 *n*	(%)	Controls^b^ N = 21297 *n*	(%)	Unadjusted OR^c^ (95% CI)	Adjusted OR^d^ (95% CI)
No use^e^	1783	(83.8)	17908	(84.1)	1.00 (Reference)	1.00 (Reference)
Paroxetine
1–23	69	(3.2)	629	(3.0)	1.10 (0.86, 1.42)	1.09 (0.85, 1.41)
≥24	15	(0.7)	131	(0.6)	1.15 (0.67, 1.97)	1.13 (0.66, 1.93)
Sertraline
1–23	37	(1.7)	295	(1.4)	1.26 (0.89, 1.78)	1.26 (0.89, 1.78)
≥24	12	(0.6)	66	(0.3)	1.83 (0.99, 3.39)	1.83 (0.99, 3.40)
Fluoxetine
1–23	48	(2.3)	613	(2.9)	0.79 (0.58, 1.06)	0.79 (0.58, 1.06)
≥24	14	(0.7)	167	(0.8)	0.84 (0.49, 1.46)	0.83 (0.48, 1.44)

*OR, odds ratio(s); CI, confidence intervals*.

*^a^SSRI (selective serotonin reuptake inhibitor) use anytime during follow-up 2 or more years prior to index date where the number of prescriptions dispensed approximates the number of months exposed*.

*^b^151 cases and 1488 controls who were not exclusive users of one agent were not included in this analysis*.

*^c^Matched on age in 5-year age groups but unadjusted for the influence of other risk factors*.

*^d^Adjusted for age at index (years), marital status, income support status, residence status, and use of oral contraceptives and/or use of hormone therapy*.

*^e^“No use” (reference category) refers to women with no SSRI prescriptions dispensed 2 or more years prior to index date. Subjects may have filled prescription(s) for SSRIs during the 2 years prior to index date which was not considered to be etiologically relevant*.

*Percentages may not add up to 100% due to rounding*.

Findings were unaffected for all analyses after changing the referent category from no prescriptions to ≤1 prescription.

## Discussion

In this large population-based case-control study, we found no evidence of increased risk of breast cancer associated with the use of high inhibitor SSRI antidepressants regardless of their duration of use or menopausal status. While we cannot rule out the possibility of a clinically important risk increase for long-term users of sertraline (≥24 prescriptions), given the small number of exposed cases (*n* = 12), the borderline statistical significance and the wide confidence interval, these results need to be interpreted cautiously.

Our study is one of the largest studies published to date, and it is the first to categorize SSRI exposure on the basis of the degree of serotonin reuptake inhibition and thus, potential impact on prolactin levels. As such, it is difficult to compare our results to those of previous studies. Generally, our findings are consistent with those studies that evaluated the relationship between SSRI use and breast cancer risk (Coogan et al., 2005, 2008; Gonzalez-Perez and Garcia Rodriguez, 2005; Chien et al., 2006; Fulton-Kehoe et al., 2006; Ashbury et al., 2010; Cosgrove et al., 2011). In addition, our results of no increased risk regardless of menopausal status are consistent with previous studies that examined this relationship including one that used the same age-based definition for pre- and post-menopausal status as in our study (Gonzalez-Perez and Garcia Rodriguez, 2005) and two other studies which defined menopausal status based on menstrual history data obtained from in-person interviews (Coogan et al., 2005, 2008).

While the point estimates for exclusive use of each of the three individual high inhibitors (paroxetine, sertraline, and fluoxetine) appear to be different, any comparisons between results for these three drugs are limited due to overlapping 95% confidence intervals and small numbers of exposed cases (reflected in the relatively wide confidence intervals). Other studies that assessed risk of breast cancer according to duration of sertraline use did not observe a statistically significant increase in risk associated with longer durations of use (Coogan et al., 2005; Chien et al., 2006; Fulton-Kehoe et al., 2006; Wernli et al., 2009; Ashbury et al., 2010). However, similar to our analysis, these studies were limited by a small number of long-term users of this agent and corresponding wide confidence intervals. In addition, our findings were consistent with other observational studies that specifically assessed breast cancer risk associated with long-term use of paroxetine (Gonzalez-Perez and Garcia Rodriguez, 2005; Haque et al., 2005; Chien et al., 2006; Fulton-Kehoe et al., 2006; Ashbury et al., 2010) and fluoxetine (Coogan et al., 2005; Gonzalez-Perez and Garcia Rodriguez, 2005; Chien et al., 2006; Fulton-Kehoe et al., 2006; Wernli et al., 2009; Ashbury et al., 2010) with the exception of Wernli et al. (2009) who reported a non-significant dose response decrease in risk associated with increasing duration of paroxetine use.

Our findings do not support a serotonin-mediated pathway for the prolactin-breast cancer hypothesis even after taking into account the extent of serotonin reuptake inhibition of individual SSRIs and the duration of their use. Studies have reported varied elevations in basal circulating prolactin measures with SSRI use – often minimal to moderate increases in prolactin levels that are not necessarily classified as hyperprolactinemia (Emiliano and Fudge, 2004; Molitch, 2005; Madhusoodanan et al., 2010; Trenque et al., 2011). Therefore it is possible that chronic modest increases in baseline circulating prolactin levels associated with the use of high inhibitor SSRIs may not exceed the threshold necessary to promote breast cancer development. Further, current evidence suggests that the synthesis and secretion of prolactin is not solely regulated by the pituitary gland, leading to questions about the carcinogenic role of circulating prolactin (Clevenger et al., 2003; Fernandez et al., 2010). Prolactin produced in breast epithelium independent of circulating prolactin may regulate cell growth and differentiation in breast tissue (Clevenger et al., 2003). Therefore higher levels of prolactin secreted by the pituitary gland secondary to an SSRI-mediated increase in serotonin may not represent the most relevant route of prolactin exposure at the tissue level in relation to breast cancer etiology (Tworoger and Hankinson, 2008). Alternatively, our reliance on the dissociation constant (Kd) for grouping SSRIs as high and lower inhibitors may have introduced misclassification of their effect on prolactin levels if the Kd measure was not a good approximation of the prolactin-enhancing potential of each SSRI.

The limitations of our study need to be considered. First, as this study relied solely on administrative health and cancer registry data, the analysis did not account for some established risk factors for breast cancer including family history, menstrual and reproductive history. Studies have indicated that users of antidepressants as a class have higher BMI, more reproductive risk factors, positive family history of breast cancer, increased likelihood of a recent mammogram, and higher rates of HT or OC use than non-users of antidepressants (Moorman et al., 2003; Chien et al., 2006; Fulton-Kehoe et al., 2006). If these sources of confounding had influenced our results, our estimates would have been spuriously elevated. As we found no association, residual confounding cannot explain our results. Further, studies of physician prescribing practices have not reported associations with the aforementioned risk factors (Olfson et al., 1998; Mojtabai, 2002; Sleath and Shih, 2003). In addition, the analysis did not account for use of other antidepressant medications such as tricyclic antidepressants (TCAs). However, most previous studies of TCAs have reported no association with breast cancer risk (Chien et al., 2006; Fulton-Kehoe et al., 2006; Wernli et al., 2009).

Also, the low prevalence of long-term use of SSRIs in our study population resulted in underpowered analyses of the safety of individual agents. However, our primary analysis of breast cancer risk according to the degree of inhibition of serotonin reuptake, a proxy for their impact on prolactin levels, was fully powered. Also, given evidence suggesting that SSRIs are often prescribed for relatively short periods of time (1–2 years) (Rosholm et al., 1997), and that 50–80% of SSRI users discontinue their treatment after less than 1 year (Meijer et al., 2004; Aikens et al., 2005) we postulate that designing studies with larger sample sizes of long-term SSRI users may not be feasible due to the challenges of obtaining a sufficient number of long-term users within the context of past and current SSRI prescribing trends. Finally, our results may not be generalizable to populations with a higher prevalence of long-term use of SSRIs or longer duration of use as only 4% of women in our study had received ≥24 prescriptions and the median estimated duration of use was 7 months (inter quartile range = 2–24 months).

In conclusion, we found no evidence of an increased risk of breast cancer associated with the use of SSRIs even after accounting for their degree of serotonin reuptake inhibition and their duration of use in women aged 28–79. Our results do not support the serotonin-mediated pathway for the prolactin-breast cancer hypothesis.

## Conflict of Interest Statement

The authors declare that the research was conducted in the absence of any commercial or financial relationships that could be construed as a potential conflict of interest.
